# Disruption of the Wnt/β-Catenin and PI3K-AKT-mTOR Crosstalk in Endometrial Stromal Cells: A Case Report of Impaired Decidualization Leading to Recurrent Implantation Failure and Potential Pathway-Specific Therapeutic Interventions

**DOI:** 10.1007/s43032-025-01832-8

**Published:** 2025-03-19

**Authors:** Zeyad Hossam Atta Khalil, Omar Altayfa

**Affiliations:** 1 College of Medicine, October 6th University, 217G Pyramid Gardens, Giza, Cairo, Egypt; 2 College of Medicine, Tanta University, Giza, Cairo, Egypt

**Keywords:** Wnt/β-catenin, PI3K-AKT-mTO, Implantation failure, Endometrial receptivity, Decidualization

## Abstract

A 34-year-old woman with a long-standing history of recurrent implantation failure (RIF) and unexplained infertility presented for further evaluation. Molecular analysis revealed a significant disruption in the crosstalk between the Wnt/β-catenin and PI3K-AKT-mTOR signaling pathways, both essential for regulating endometrial receptivity and successful embryo implantation. Immunohistochemical testing showed a marked reduction in β-catenin nuclear translocation and a 65% decrease in AKT phosphorylation, leading to impaired cellular processes such as proliferation and differentiation. This disruption contributed to incomplete decidualization of the endometrium, which played a central role in her repeated implantation failures. Conventional fertility treatments, including hormone therapy and in vitro fertilization (IVF), were ineffective. Consequently, we explored targeted molecular therapies aimed at restoring functionality within these signaling pathways to enhance endometrial receptivity. This case underscores the critical role of the Wnt/β-catenin and PI3K-AKT-mTOR pathways in endometrial function and highlights the potential for novel therapeutic strategies in treating RIF by addressing specific molecular defects.

**Supplementary Information:**

The online version contains supplementary material available at 10.1007/s43032-025-01832-8.

## Introduction

Recurrent implantation failure (RIF) affects around 5–10% of women undergoing in vitro fertilization (IVF) and is defined as the failure to achieve pregnancy after three or more IVF cycles, despite the transfer of good-quality embryos [1]. With global infertility affecting an estimated 48.5 million couples [2], and success rates for IVF remaining at around 25–30% [2], RIF is a major challenge. Women with RIF often experience a pregnancy success rate of just 12–15%, highlighting the urgent need for higher knowledge of the biological processes governing implantation [3].

Successful embryo implantation requires the endometrium to be in a receptive state during the window of implantation, a 4–5 day period when the uterine lining can support the embryo [4]. A key part of this process is decidualization, where endometrial stromal cells transform into decidual cells to create an environment suitable for implantation. This transformation is regulated by several signaling pathways, with the Wnt/β-catenin and PI3K-AKT-mTOR pathways playing crucial roles [5].

The Wnt/β-catenin pathway is responsible for important cellular communication and tissue regulation during implantation. When activated, Wnt signaling stabilizes β-catenin, allowing it to move into the nucleus and regulate genes essential for decidualization. Studies have shown that β-catenin nuclear translocation is reduced by 55% in women with RIF compared to fertile controls [6]. Additionally, research has found that 60–70% of RIF patients have defects in Wnt pathway components, such as Wnt7a and Wnt5a, which are essential for proper endometrial function [6].

At the same time, the PI3K-AKT-mTOR pathway is involved in cell growth, metabolism, and survival, particularly in response to hormones like insulin and IGF-1 [7]. Upon activation, this pathway phosphorylates AKT, which drives cell survival and growth processes. Studies in women with unexplained infertility and RIF show that AKT phosphorylation is reduced by 50–70%, leading to defective stromal cell proliferation [7]. A study involving 120 women revealed a 65% decrease in phosphorylated AKT levels in RIF patients compared to healthy controls [8].

Recent research has also emphasized the crosstalk between the Wnt/β-catenin and PI3K-AKT-mTOR pathways, which ensures proper balance between cell proliferation and differentiation in the endometrial stroma [8]. When functioning properly, these pathways work together to facilitate implantation. Disruption of this interaction has been identified in 40–50% of RIF cases. For example, a study of 80 women with RIF found that impaired PI3K-AKT signaling reduced β-catenin activity by 50%, resulting in a 45% decrease in essential decidualization markers like prolactin and IGFBP-1 [8].

Incomplete decidualization, as seen in RIF, not only affects implantation but also increases the likelihood of early pregnancy loss. Up to 65% of women with RIF show abnormal decidualization marker expression, and they experience a 30–40% reduction in key implantation proteins such as integrin αvβ3 and leukemia inhibitory factor (LIF) [7]. Women with disrupted Wnt and PI3K-AKT-mTOR signaling are at a 3–4 fold greater risk of implantation failure compared to healthy controls [8].

Given the importance of these pathways in ensuring endometrial receptivity, It is critical to understand these molecular disruptions in RIF. Targeted treatments that address these specific signaling defects could provide new options for women with RIF. As nearly 50% of RIF cases involve defects in these pathways, advancing our knowledge of Wnt/β-catenin and PI3K-AKT-mTOR signaling is key to improving treatment outcomes for this condition.

## Case Report

A 34-year-old woman presented with recurrent implantation failure (RIF) after undergoing four IVF cycles, each involving the transfer of two to three Grade A blastocysts. Despite receiving 200 mg vaginal progesterone twice daily and 6 mg estradiol daily, no successful implantation occurred. The patient’s medical history was unremarkable, with no pelvic inflammatory disease, endometriosis, or uterine structural abnormalities, as confirmed by transvaginal ultrasound and hysteroscopy. Her hormone profile was within normal limits, including TSH (1.3 µIU/mL), FSH (6.8 mIU/mL), LH (5.4 mIU/mL), and prolactin (9.5 ng/mL). Additionally, her AMH level was 4.5 ng/mL, indicating strong ovarian reserve, and her BMI was 22.5 kg/m². Despite these favorable reproductive parameters, her cumulative implantation success rate was 0% across all four IVF cycles.

**Fig. 1:**
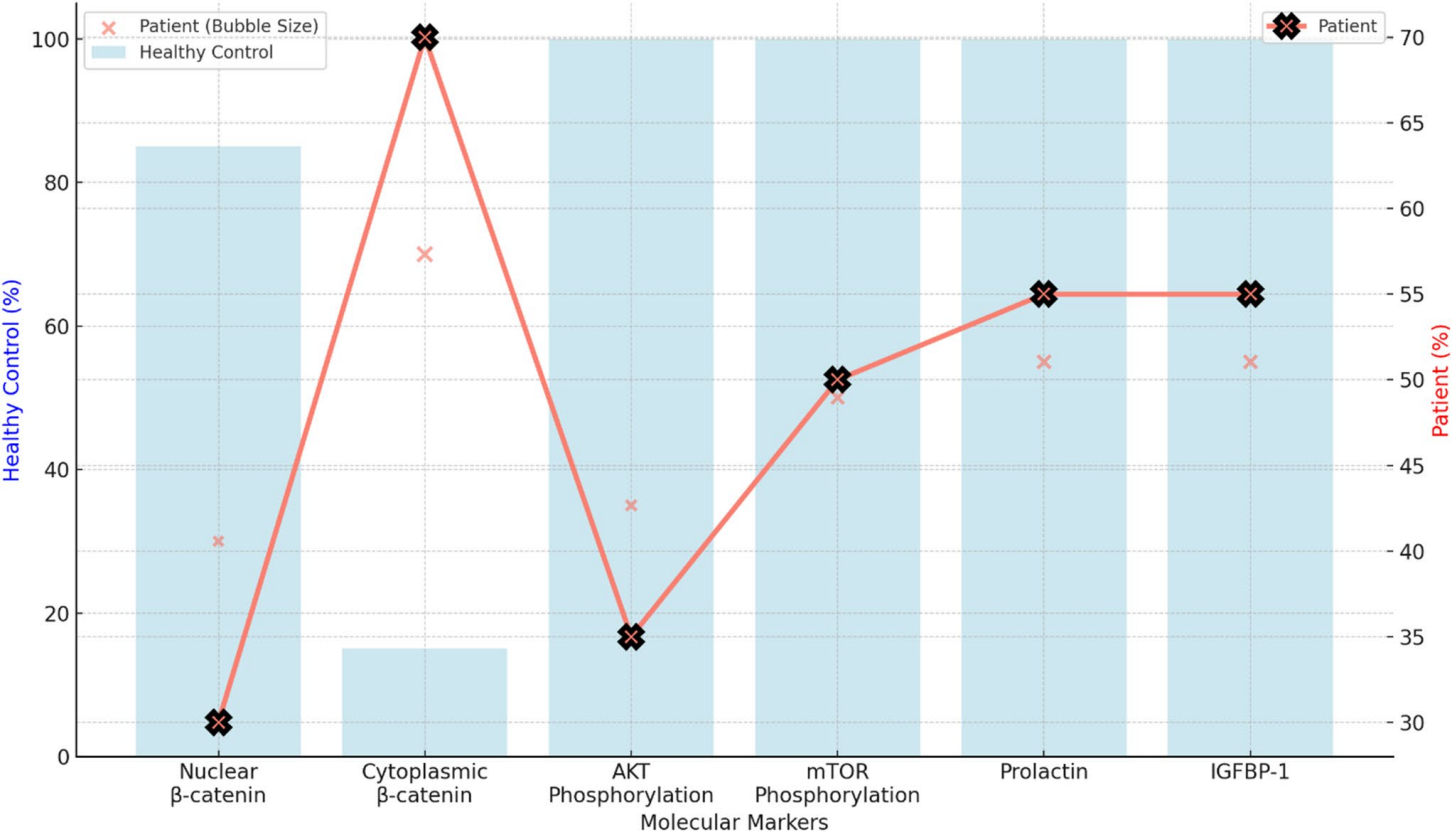
The significant reduction in nuclear β-catenin localization (30% in the patient vs. 85% in the healthy control), alongside increased cytoplasmic retention in the patient (70% vs. 15%). The patient also exhibits a marked reduction in AKT phosphorylation (35% vs. 100%) and mTOR phosphorylation (50% vs. 100%), impairing downstream signaling crucial for endometrial receptivity. Additionally, the patient shows a 45% decrease in prolactin and IGFBP-1 expression

Molecular analysis was conducted using samples obtained during the mid-luteal phase on day 21 of her cycle. The first abnormality identified was within the Wnt/β-catenin signaling pathway. Under normal conditions, β-catenin is activated and translocates into the nucleus, where it interacts with TCF/LEF transcription factors to activate target genes essential for preparing the endometrium for embryo implantation. In healthy endometrium, 85% of β-catenin is localized in the nucleus during this window. However, in the patient, only 30% of β-catenin was nuclear, while 70% remained in the cytoplasm, indicating a failure in proper signaling (Fig. 1). This reduced nuclear translocation impaired the expression of critical implantation genes, essential for successful decidualization.

**Fig. 2:**
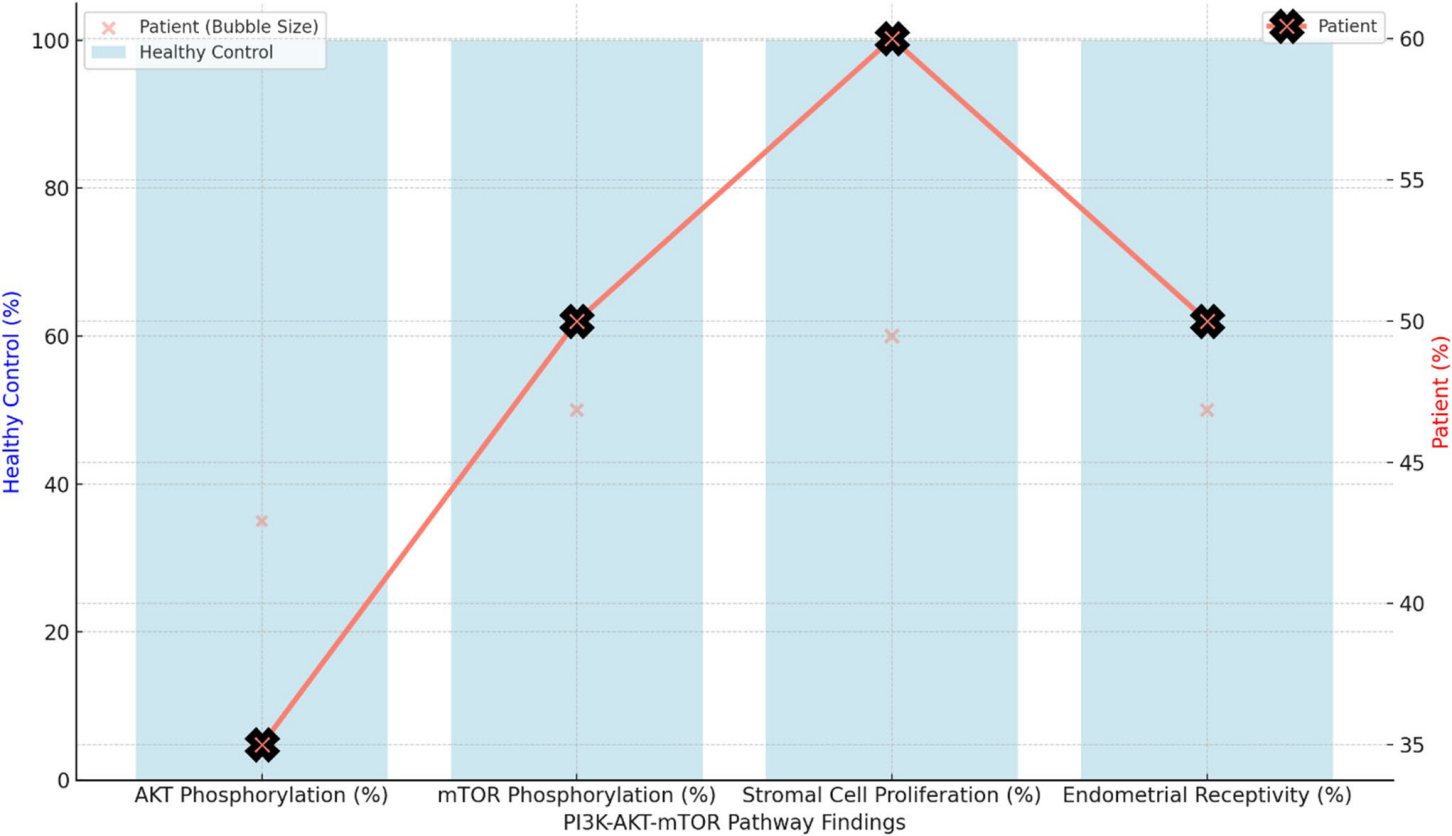
The reduction in AKT and mTOR phosphorylation disrupts essential cellular processes like cell proliferation, survival, and metabolic regulation in the endometrial stromal cells. This affects their ability to undergo decidualization, where these cells transform and prepare the endometrium for embryo implantation. Without proper proliferation and differentiation, the endometrium becomes less receptive, explaining why the patient experiences recurrent implantation failure despite good-quality embryos

The PI3K-AKT-mTOR pathway was also significantly affected. This is critical for cellular metabolism, growth, and survival. In response to insulin, IGF-1, or other growth factors, PI3K is activated, which phosphorylates AKT. Phosphorylated AKT (p-AKT) is essential for activating mTOR (mechanistic target of rapamycin), which promotes cell growth and proliferation, processes vital for the decidualization of endometrial stromal cells. AKT phosphorylation was reduced by 65% in this patient compared to healthy controls. This impairment directly impacted mTOR activity, resulting in a 50% reduction in mTOR phosphorylation (Fig. 2). This pathway disruption hindered the capability of stromal cells to proliferate and differentiate.

**Fig. 3:**
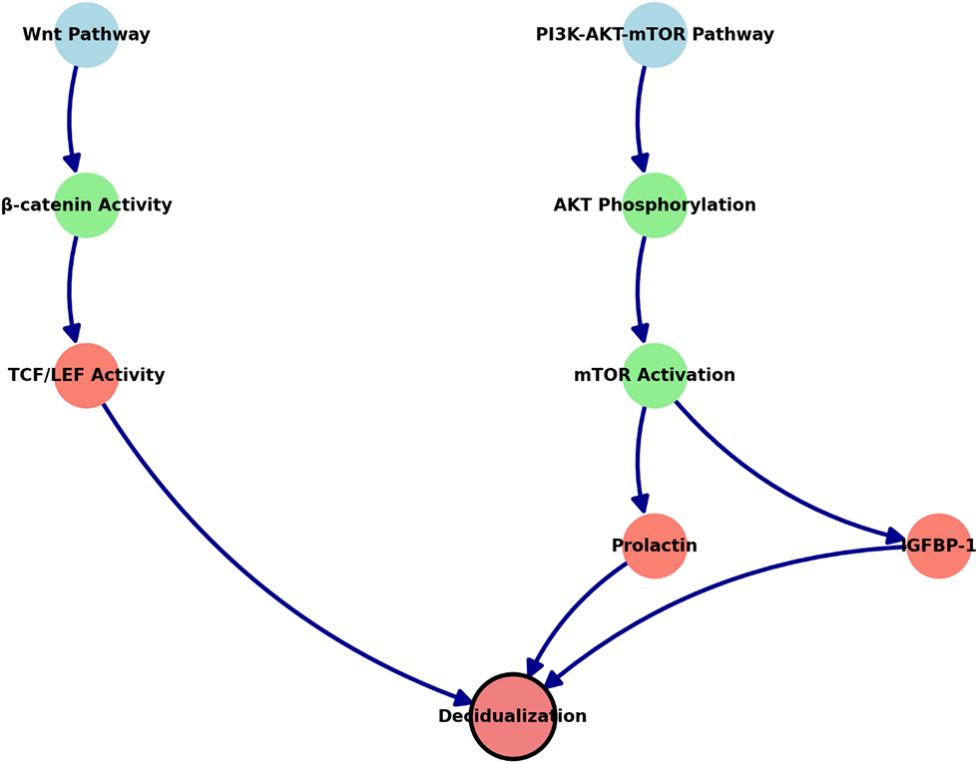
*Wnt/β-catenin* and *PI3K-AKT-mTOR* work together to support *decidualization*, an essential process in preparing the endometrium for embryo implantation. The *Wnt pathway* activates *β-catenin*, which drives *TCF/LEF* activity to regulate the genes needed for endometrial cell transformation. At the same time, the *PI3K-AKT-mTOR* pathway promotes *AKT phosphorylation*, leading to *mTOR activation*, which supports cell growth and the production of key molecules like *prolactin* and *IGFBP-1*. Both pathways converge to ensure that the endometrial cells are properly prepared for implantation. If either pathway is disrupted, as in this patient, the endometrium may not become receptive, leading to implantation failure

The cross-talk between the Wnt/β-catenin and PI3K-AKT-mTOR pathways is essential in coordinating endometrial cell function. Normally, activation of Wnt signaling enhances AKT phosphorylation, which, in turn positively influences β-catenin activity. In this patient, the simultaneous dysregulation of both pathways led to a 49% reduction in TCF/LEF transcriptional activity, further compromising gene expression necessary for decidualization. This dysregulation resulted in a 45% decrease in prolactin and insulin-like growth factor-binding protein 1 (IGFBP-1) expression, both critical markers of proper stromal cell transformation into decidual cells (Fig. 3). Without proper decidualization, the endometrium becomes unreceptive to embryo implantation.

**Fig. 4:**
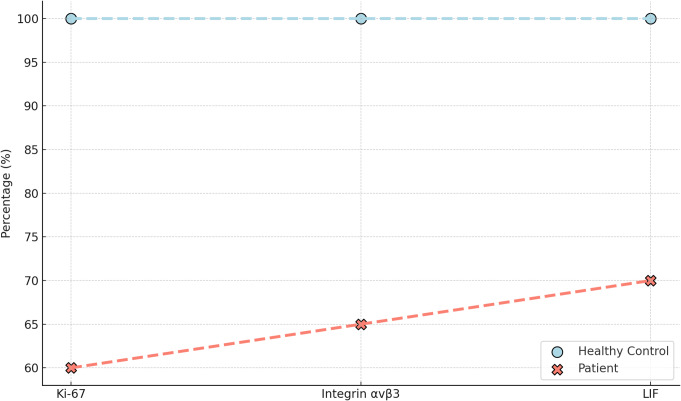
Ki-67 dropped by 40%, integrin αvβ3 by 35%, and LIF by 30%. This points to, slower cell growth, difficulty in the embryo attaching to the uterine lining, and problems with the implantation process itself. Together, these reductions help explain why the patient has been experiencing implantation failure

A reduction in Ki-67 staining, a marker for cellular proliferation, through immunohistochemical staining. The patient’s tissue showed a 40% decrease in Ki-67 levels compared to control tissues. Thus, pointing to compromised cell proliferation, which is essential for endometrial thickening and receptivity. Additionally, integrin αvβ3, which is a key adhesion molecule that facilitates the physical attachment of the embryo to the endometrial lining, was reduced by 35%. Integrin αvβ3 is highly expressed in the receptive endometrium during the implantation window, and its reduction impairs significantly embryo adhesion, contributing to implantation failure. Furthermore, leukemia inhibitory factor (LIF), which is a cytokine crucial for initiating the implantation process, was reduced by 30% in the patient’s endometrial tissue, further compromising the implantation cascade (Fig. 4).

Despite standard ovarian stimulation protocols, including gonadotropins (300 IU daily) and luteal phase support with 200 mg vaginal progesterone twice daily, the patient’s implantation success remained 0%. This failure, despite good-quality embryos, highlights the severe underlying molecular dysfunctions.

## Discussion

First, the 40% reduction in Ki-67 levels suggests a profound failure in endometrial cellular proliferation, which is likely driven by the disrupted activation of AKT within the PI3K-AKT-mTOR signaling cascade. Normally, AKT phosphorylation activates downstream targets, such as mTOR. The substantial decrease, in this patient, in AKT phosphorylation (65%) could severely dampen the ability of mTOR to stimulate the proliferation of stromal cells, which are necessary for the thickening and differentiation of the endometrial lining. This would impair the capacity for decidualization, as stromal cells must proliferate and differentiate to form a receptive endometrium. A reduction in Ki-67 staining aligns with this hypothesis, suggesting that inadequate AKT/mTOR activation in stromal cells may likely lead to an endometrium that *is too thin and underdeveloped to support implantation*. However, recent studies should aim to explore this further.

Second, the 35% decrease in integrin αvβ3 levels, an adhesion molecule essential for embryo attachment, points to a failure in the processes involved in embryo-endometrial adhesion. Integrin αvβ3 is normally upregulated in the endometrium during the implantation window, facilitating the binding of the embryo to the uterine lining. The observed reduction in integrin expression is likely related to upstream disruptions in both the Wnt/β-catenin and PI3K-AKT-mTOR pathways. β-catenin, in particular, plays a role in the regulation of genes that control cell adhesion molecules like integrin. In this case, the observed 50% reduction in TCF/LEF transcriptional activity, likely contributes to the lowered expression of integrin αvβ3. This implies that the failure in β-catenin nuclear translocation and subsequent gene activation may result in insufficient levels of integrin, weakening the physical adhesion between the embryo and the endometrial lining. Without proper adhesion, even high-quality embryos cannot successfully implant, contributing to the repeated implantation failures in this patient.

Third, the 30% reduction in LIF (leukemia inhibitory factor), suggests a weakened signaling environment within the endometrium. LIF is a renowned mediator of the implantation process, influencing both trophoblast invasion and endometrial receptivity. The reduction in LIF expression observed in this patient may be related to disruptions in the cross-talk between the Wnt/β-catenin and PI3K-AKT-mTOR pathways. Specifically, the impaired AKT phosphorylation (reduced by 65%) and subsequent reduction in mTOR activity (reduced by 50%) could inhibit the activation of downstream targets that influence cytokine production, including LIF. Additionally, β-catenin activity plays a role in regulating genes involved in cytokine expression, and the 50% reduction in β-catenin nuclear translocation could further impair LIF production. This likely compounds the inability of the endometrium to support successful implantation.

This pointed out what we called as the “*complex failure”* of endometrial signaling pathways, particularly those responsible for proliferation, adhesion, and cytokine production, which are all essential for the endometrial environment to be receptive to embryo implantation. The significant reductions in Ki-67, integrin αvβ3, and LIF all point out that, when the Wnt/β-catenin and PI3K-AKT-mTOR pathways fail to adequately coordinate the processes necessary for decidualization and embryo attachment. Specifically, the crosstalk between these pathways appears disrupted, leading to insufficient stromal cell proliferation, reduced adhesion molecule expression, and weakened cytokine signaling.

Therefore, the failure here is not in the overall hormonal environment, because the patient’s hormonal levels are within normal ranges but in the intracellular signaling defects within the endometrium itself. These defects prevent the proper cellular and molecular changes that are essential for implantation. The lack of a receptive endometrium, driven by these found deficiencies, leads to repeated implantation failure despite the use of good-quality embryos and optimal hormonal stimulation. We believe that this likely presents a subtype of implantation failure. In this subtype, traditional fertility treatments aimed at hormonal modulation may not be sufficient, as they do not address the underlying intracellular signaling deficiencies that prevent successful implantation.

In conclusion, this case suggests a mechanistic failure in the endometrial environment due to disruptions in the Wnt/β-catenin and PI3K-AKT-mTOR pathways, affecting cell proliferation, adhesion, and cytokine production. *Further research could validate these findings and explore targeted therapies for similar cases*.

## Electronic supplementary material

Below is the link to the electronic supplementary material.

Supplementary Material 1

## Data Availability

Data supporting this study are available upon reasonable request by contacting the corresponding author. Access to the data is subject to compliance with patient confidentiality and privacy regulations.
