# Supplementary material for: Disruption of the Wnt/β-Catenin and PI3K-AKT-mTOR Crosstalk in Endometrial Stromal Cells: A Case Report of Impaired Decidualization Leading to Recurrent Implantation Failure and Potential Pathway-Specific Therapeutic Interventions
Source: Reprod Sci. 2025 Mar 19;32(6):1953–8. doi: 10.1007/s43032-025-01832-8 (PMC12187874; doi:10.1007/s43032-025-01832-8)
Supplement: Supplementary file 1 — Supplementary Material 1 [file 43032_2025_1832_MOESM1_ESM.docx]

**لسيد/ة المحترم/ة،**

**تحية طيبة وبعد،**

**نود إعلامكم بأن هذه الدراسة قد تم تنفيذها وفقًا للمعايير الأخلاقية المتبعة في لجان البحث المؤسسية والوطنية، وكذلك وفقًا لإعلان هلسنكي وتعديلاته اللاحقة أو المعايير الأخلاقية المقارنة. تم الحصول على الموافقة الأخلاقية من لجنة مراجعة الأبحاث المؤسسية (IRB) في مستشفى جامعة 6 أكتوبر، برقم الموافقة O6U-IRB-2024-058. كما تم الحصول على الموافقة المستنيرة كتابيًا من المريض قبل إدراجه في الدراسة، مع التأكيد على سرية المعلومات والمشاركة الطوعية.**

**نود أيضًا توضيح أن د. محمد حافظ هو الذي قام بتقديم طلب الموافقة والحصول عليها من لجنة مراجعة الأبحاث في المستشفى.**

**شكرًا لتفهمكم وتعاونكم.**

**وتفضلوا بقبول فائق الاحترام والتقدير،**

**مستشفى جامعة 6 أكتوبر**

**IRB Approval**

Dear Sir/Madam,

We would like to inform you that this study was conducted in accordance with the ethical standards set by institutional and national research committees, as well as the Helsinki Declaration and its later amendments or comparable ethical standards. Ethical approval was obtained from the Institutional Review Board (IRB) of October 6th University Hospital, with the approval number O6U-IRB-2024-058. Written informed consent was secured from the patient prior to their inclusion in the study, ensuring confidentiality and voluntary participation.

Additionally, we would like to clarify that Dr. Mohammed Hafez was responsible for filing the approval request and obtaining the necessary consent from the IRB at the hospital.

Thank you for your understanding and cooperation.

Kind regards,
October 6th University Hospital
